# The Putative AKH Receptor of the Tobacco Hornworm, *Manduca sexta*, and Its Expression

**DOI:** 10.1673/031.011.0140

**Published:** 2011-04-05

**Authors:** R. Ziegler, J. Isoe, W. Moore, M. A. Riehle, M. A. Wells

**Affiliations:** ^1^ Department of Entomology, The University of Arizona, Tucson, AZ 85721 USA; ^2^ Department of Chemistry and Biochemistry, The University of Arizona, Tucson, AZ 85721 USA

**Keywords:** Adipokinetic hormone, adipokinetic hormone receptor, fat body, mRNA expression, Real Time PCR

## Abstract

Adipokinetic hormones are peptide hormones that mobilize lipids and/or carbohydrates for flight in adult insects and activate glycogen Phosphorylase in larvae during starvation and during molt. We previously examined the functional roles of adipokinetic hormone in *Manduca sexta* L. (Lepidoptera: Sphingidae). Here we report the cloning of the full-length cDNA encoding the putative adipokinetic hormone receptor from the fat body of *M. sexta.* The sequence analysis shows that the deduced amino acid sequence shares common motifs of G protein-coupled receptors, by having seven hydrophobic transmembrane segments. We examined the mRNA expression pattern of the adipokinetic hormone receptor by quantitative Real-Time PCR in fat body during development and in different tissues and found the strongest expression in fat body of larvae two days after molt to the fifth instar. We discuss these results in relation to some of our earlier results. We also compare the *M. sexta* adipokinetic hormone receptor with the known adipokinetic hormone receptors of other insects and with gonadotropin releasing hormone-like receptors of invertebrates.

## Introduction

The adipokinetic hormone (AKH) is an insect peptide hormone produced in and released from the corpora cardiaca (CC) (Ziegler et al. 1988; Van der Horst 2003). In some insects, in which it controls the mobilization of carbohydrates, it is called hypertrehalosemic hormone.

Many different forms of this peptide hormone have been described in different insects (Gaede et al. 1994), and AKH appears to be present in all insects. AKH was first described in locusts to control the mobilization of fat body lipids for flight (Mayer and Candy 1969; Beenakkers 1969). It has the same function in other insects that use lipids as an energy source for flight, such as *Manduca sexta* L. (Lepidoptera: Sphingidae) (Ziegler and Schulz 1986). In *Drosophila melanogaster* AKH controls carbohydrate and lipid homeostasis (Lee and Park 2004; Groenke et al. 2007; Bharucha et al. 2008). Besides mobilizing energy reserves, AKH inhibits RNA synthesis (Kodrik and Goldsworthy 1995), protein and lipid synthesis (Gokuldas et al. 1988; Ziegler 1997) and it stimulates locomotory activity in *Pyrrhocoris apterus* (Socha et al. 1999). AKH has also been shown to have a role in the immunity of *Locusta migratoria* (Goldsworthy et al. 2005).

AKH, being a peptide hormone, acts through a G protein-coupled membrane receptor with seven transmembrane segments. Recently the AKH receptors of *Bombyx mori* and of *D. melanogaster* have been cloned (Staubli et al. 2002), as well as the AKH receptor of *Periplaneta americana* (Hansen et al.2006; Wicher et al. 2006), and of *Anopheles gambiae* (Kaufmann and Brown 2006, Belmont et al. 2006). The AKH receptor of *Apis mellifera, Tribolium castaneum, Aedes aegypti, Acyrthosiphon pisum, Pediculus humanus corporis* and *Nasonia vitripennis* have been deduced from their genomic sequences. Tissue-specific expression studies of the mRNA of the AKH receptor have been performed in *P. americana* and *A. gambiae* (Wicher et al. 2006; Kaufmann and Brown 2006). Developmental changes in the expression of AKH receptors have been performed with RT-PCR in *A. gambiae* (Kaufmann and Brown 2006) and in *Ae. aegypti* (Kaufmann et al. 2009).

We previously demonstrated in *M. sexta,* that AKH mobilizes lipids for flight in the adults (Ziegler and Schulz 1986), and that in larvae, it activates fat body glycogen Phosphorylase (GP) during molt and starvation (Siegert and Ziegler 1983; Siegert 1988; Gies et al. 1988; Ziegler et al. 1990). The activation of GP during starvation does not occur if the CC, the source of AKH, are surgically removed, indicating that starvation induces the release of AKH from the CC which in turn activates GP of fat body (Siegert and Ziegler 1983). AKH injected into larvae of different ages during the last instar activates GP of fat body with age-dependent intensity (Ziegler 1984). Differences in the response seen could be due to changes in the amount of the AKH receptor present in fat body.

In this paper we report the cloning of the full-length cDNA encoding the putative AKH receptor from *M. sexta,* and we report fluctuations of the AKH receptor mRNA in fat body during the final larval instar, during pharate adult life, and during the early days of adult life. We also examined the expression of AKH receptor mRNA in different tissues of *M. sexta.* We confirm the identity of this gene and explore its evolution within insects by inferring the gene tree with Bayesian inference methods.

## Materials and Methods

### Animals

Tobacco hornworms, *M. sexta,* were reared according to the rearing techniques of Bell and Joachim (1976), with minor modifications. The colony was originally established from eggs obtained from USDA, State University Station, Fargo, ND. Larvae of the fifth instar, pharate adults and adults were employed in this study.

### Cloning of the receptor

Total RNA was isolated from fat body of adult male *M. sexta* using TRIzol Reagent (Invitrogen, www.invitrogen.com) according to the supplier's instruction. Possible genomic DNA contamination was removed by DNase I (Fermentas, www.fermentas.com) treatment. From total RNA, mRNA was subsequently isolated using oligo-dT cellulose (Amersham, www.gelifesciences.com). Bioinformatic analysis of the AKH receptors of *D. melanogaster* (GenBank AAN10047) and *B. mori* (GenBank NP 001037049) showed conserved regions. Degenerate primers were designed for PCR cloning based on a stretch of amino acid residues conserved between *D. melanogaster* and *B. mori* AKH receptor. First strand cDNA synthesis was performed using a degenerate primer with the sequence 5′YTCYTTRTCDATCCA-3′ and reverse transcriptase (Promega, www.promega.com). The resulting cDNA was used as a template to isolate a fragment of the *M. sexta* AKH receptor sequence using the following degenerate primers for PCR amplification: forward 5′-GCNGGAGAYYTNATGTGYNG-3′; reverse 5′-TCYTTRTCDATCCARTACCA-3′. The amplified PCR product formed on a 1% agarose gel a single band of the expected size of 539 bp. This product was sequenced on an ABI PRISM® 377 DNA Sequencers (Applied Biosystems, www.appliedbiosystems.com) in the DNA Sequencing Facility at the Genetic Analysis and Technology Core Facility at the University of Arizona. It was 87% identical to the AKH receptor from *B. mori,* indicating that a nucleotide sequence encoding part of the *M. sexta* AKH receptor was cloned. The nucleotide sequence from this clone was subsequently used to design *M.* sexta-specific oligonucleotide primers to determine the complete open reading frame of *M. sexta* AKH receptor by 3′ and 5′ RACE. The cDNA for 3′ RACE was synthesized from mRNA using an oligo-dT-VN primer: 5′GAACTGCAGAGGATCCACTATTTTTTTTTTTTTTTTTTVN-3′. The 3′ RACE was performed using the following forward primers: 5′-ATGATGTTCACCAGGACCTT-3′ and 5′-GGCCTCTACCTCTCCAGTT-3′ and as reverse primer: 5′GAACTGCAGAGGATCCACTA-3′. The cDNA for 5′ RACE was synthesized with StrataScript 5.0 (Stratagene, www.stratagene.com) using as primer for the first strand synthesis 5′-CACTAGGACAATCGTGACTGT-3′. The 3′ end of the first strand cDNA was tailed with terminal transferase and dATP. The second strand was synthesized with oligo-dT-VN and reverse transcriptase (Promega). The nested reverse primers for the 5′ RACE were 5′TGTCCGGGCTCTCGCTCT-3′ and 5′ACTACGACGCATCTTGTCATT-3′ and the forward primer was 5′-GAACTGCAGAGGATCCACTA-3′.

### Real-Time PCR

Quantitative Real-Time PCR (qRT-PCR) assays for *M. sexta* AKH receptor expression were performed with cDNA templates that were synthesized with random decamers from 250 ng of fat body total RNA from different developmental stages or tissues treated with DAase I (Fermentas) (nervous tissue, heart, hemocytes, flight muscle, gut, Malpighian tubules, salivary gland, testes). Total RNA was tested for DNA contamination, only in one sample from brain a minor DNA contamination was found. The cDNA was diluted 1:5 for qRT-PCR. qRT-PCR was performed with the following *M. sexta-*specific primers, forward: 5′CATACTTCCTGGTAAACATGA-3′ and reverse: 5′CACTAGGACAATCGTGACTGT-3′. The reaction mixture of 10 µl total volume contained 2 µl of cDNA, 3 µl of the primer mix (final concentration 0.3 µM) and 5 µl of SYBR Green PCR Master Mix (Applied Biosystems). qRT-PCR amplifications were carried out using a 7300 Real-Time PCR System (Applied Biosystems) in a 96-well microtiter plate. *M. sexta* ribosomal 18S rRNA was used as a standard to normalize the AKH receptor cDNA. For qRT-PCR of 18S rRNA the following primers were used, forward: 5′CCGGTAACGAACGAGACTCTA-3′ and reverse: 5′GGGCATCACAGACCTGTTATT-3′. For each developmental stage fat body or other tissues from three different animals was separately processed and samples were run in triplicate for the AKH receptor gene and 18S rRNA as an internal standard. All oligonucleotide primers were purchased from Sigma Genosys (www.sigmaaldrich.com). Data were analyzed using ABI Prism 7300 SDS Software (version 1.2.2, Applied Biosystems). A standard curve was made for both genes, and the Ct values were corrected for the inequality of the efficiencies of each pair of primers. The values for the AKH receptor were normalized with the values of the corresponding expression level of the internal standard 18S rRNA. Dissociation curve analysis was performed to ensure the specificity of each amplicon.

### Phylogenetic Methods

The amino acid sequences of all known insect AKH receptors were compared. The sequence of human gonadotropin releasing hormone receptor was included in the comparison as AKH receptors have similarity with human gonadotropin releasing hormone receptor. In addition the non-vertebrate orthologues from the water flea *Daphnia pulex,* Pacific oyster *Crassostrea gigas, Octopus vulgaris,* the sea urchin *Strongylocentrotus purpuratus,* the sea squirt *Ciona intestinalis,* the lancelet *Branchiostoma floridae,* the roundworm *Caenorhapditis elegans,* and the Placozoan *Trichoplax adherens* were included. They are cited in GenBank as gonadotropin releasing hormone receptors. Identities of these sequences were assessed using the Needleman-Wunsch Global Sequence Alignment Tool available on the National Center for Biotechnology Information (NCBI) website.

Initial alignments were created using Opal (Thompson and Higgins 1994) and adjusted manually and converted into amino acids in Mesquite 2.72 (Maddison and Maddison 2010). ProtTest 2.0 (Drummond and Strimmer 2001; Guindon and Gascuel 2003; Abascal et al. 2005) was used to select the model of protein evolution that best fit the alignment. The model chosen by both the Akaike Information Criterion (AIC) and the Bayesian Information Criterion (BIC) was JTT + I + gamma. The posterior probability distribution of trees was approximated using the Metropolis-coupled Markov chain Monte Carlo (MC^3^) algorithm implemented in MrBayes (ver. 3.1.2; Ronquist and Huelsenbeck 2003). Each MC^3^ analysis comprised 2 simultaneous runs of 4 chains initiated from random starting trees were run for 20 million generations, with trees sampled every 1000 generations. Runs were terminated once the average standard deviation of split frequencies went below 0.01 (Huelsenbeck and Ronquist 2005). Tracer (ver.1.4; Rambaut and Drummond 2003) was used to assess chain convergence. The chains converged after 2,577,000 generations. Post burn-in trees sampled by the independent analyses were combined and summarized using the “sumt” command in MrBayes.

## Results

### Cloning of the *M. sexta* AKH receptor

**Figure 1.**
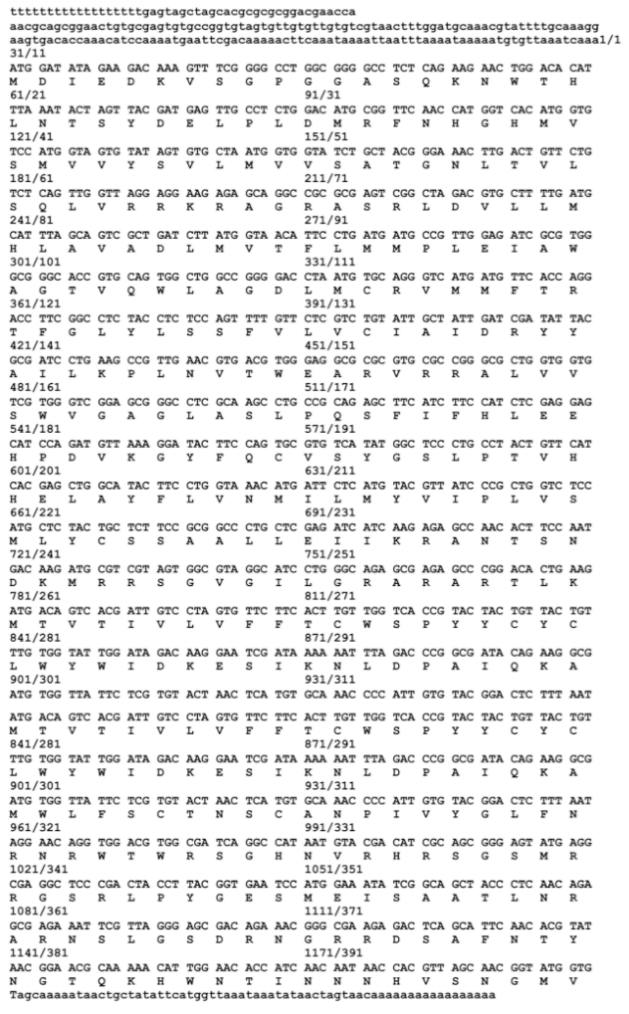
Nucleotide and the deduced amino acid sequences of the AKH receptor from *Manduca sexta.* High quality figures are available online.

**Figure 2.**
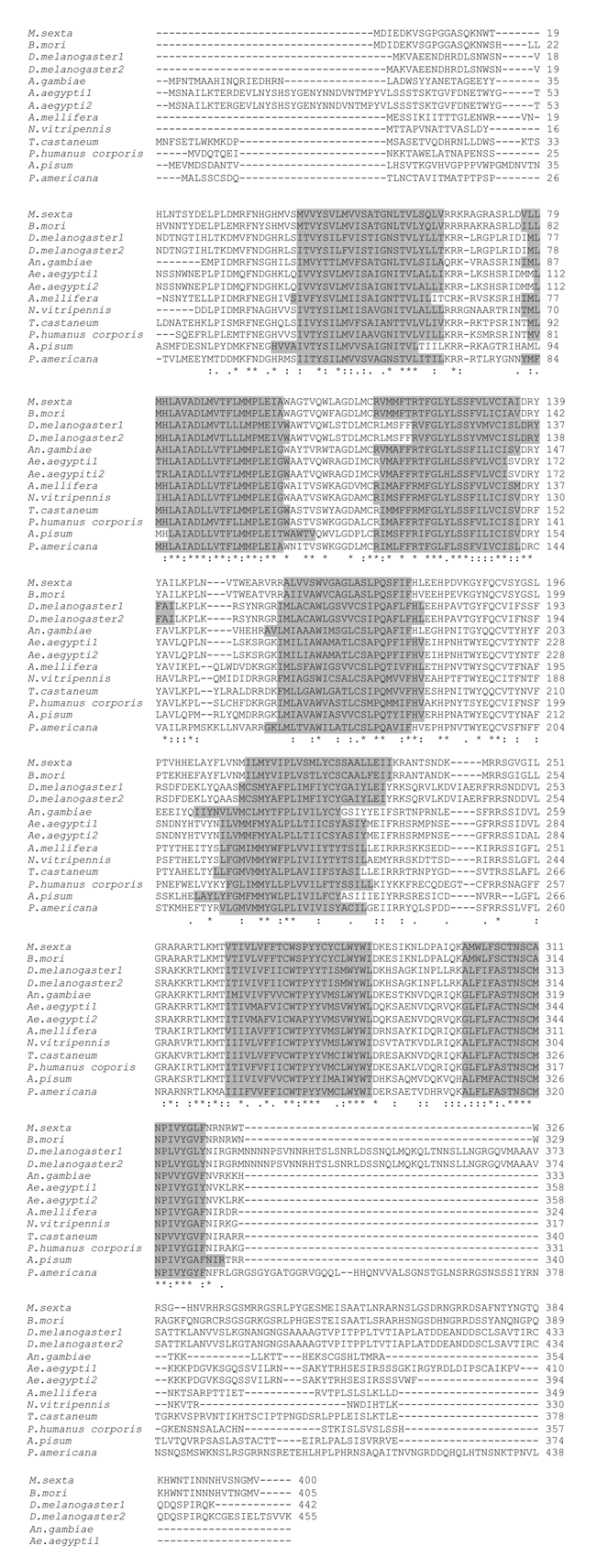
The comparison of the different AKH receptors shows that the length of the AKH receptor differs in different species between 456 (*Periplaneta americana*) and 330 amino acids (*Nasonia vitripennis*). The transmembrane segments are shaded. The main difference appears to be between the length of the N-terminal extracellular and the termninal intracellular sequence. Not only the transmembrane segments, but also the loops between the transmembrane segments have a similar length. For appreviations and GenBank accession number see legend of Figure 3. The alignment was performed with ClustalW2 EBI and the transmembrane segments were localized with TMHMM Server v.2.0 (http://www.cbs.dtu.dk/services/TMHMM-2.07). High quality figures are available online.

To clone the putative adipokinetic hormone receptor of *M. sexta,* degenerate primers were initially used, based on the conserved amino acid residues of the AKH receptor from *B. mori* and *D. melanogaster.* Subsequent 5′ and 3′ RACE resulted in the determination of a full-length AKH receptor from *M. sexta* (Figure 1) (GenBank EU440531). The *M. sexta* receptor has 400 amino acids and is predicted to generate a protein of about 45.5 kDa with an isoelectric point of 9.68. TMHMM (http://www.cbs.dtu.dk/services/TMHMM2.0/) and TopRed2 (http://www.sbc.su.se/~erikw/toppred2/), transmembrane prediction programs, predict that it is a protein with seven transmembrane segments, consistent with the AKH receptors of other insects examined (Figure 2). For *Tribolium castaneum* there are three gonadotropin releasing hormone receptors in GenBank. Only one is included here, as the other two are not AKH receptors (see discussion). For *Culex quinquefasciatus* there is one sequence of a gonadotropin—releasing hormone receptor in GenBank, but this is not an AKH receptor.

### Sequence identities

**Figure 3.**
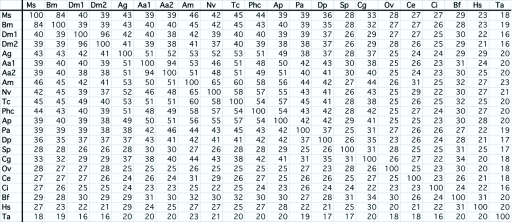
Blast results showing the percentage of identity of the sequences of the different AKH receptors, the gonadotropin releasing hormone receptor of humans and the orthologues from some non-vertebrates. A.al, Aedes *aegypti 1* (GenBank XP 001655249); A. a2, *Aedes aegypti2* (GenBank 391955.1): A.g, *Anopheles gambiae* (GenBank AAQ63187): A. m, *Apis mellifera* (GenBank AAX83121): A. p, *Acyrthosiphon pisum* (GenBank XP 001945436): B. f, *Branchiostoma floridae* (GenBank EU433377.1); B. m, *Bombyx mori* (GenBank NP 001037049): C elegans, Caenorhabditis elegans (GenBank NP 491453.1): C. g, *Crassostrea gigas* (GenBank CAP19986.1): C. i, *Ciona intestinalis* (GenBank NP 001028997.1): D. ml, *Drosophila melanogaster1* (GenBank AAN10047): D. m2, *Drosophila melanogaster2* (GenBank AAN10047.1): D. p, *Daphnia pulex* (GenBank GNO 748024): H. s, Homo sapiens (GenBank NP 491453.1): M. s, *Manduca sexta* (GenBank EU440531); N. v, *Nasonia vitripennis* (GenBank XP 001599670): O. v, *Octopus vulgaris* (GenBank AB185200). P. a, *Periplaneta americana* (GenBank ABB20590): P. hC, *Pediculus humanus corporis* (GenBank EEB 15485): S. p, Strongylocentrotus purpuratus (GenBank NP 001116990): T. a, *Trichoplax adherens* (GenBank XP 002112233.1): T. c, *Tribolium castaneum* (GenBank ABN79650 and XP 971565). High quality figures are available online.

When the amino acid sequences were analyzed by Blast (Figure 3) the receptors of *M. sexta* and *B. mori* showed high sequence identity (84% and 92% similarity), as do the receptors from the Hymenoptera *A. mellifera* and *N. vitripennis* (65%). The receptors from the Hymenoptera are also about 60% identical with the *T. castaneum* AKH receptor. The AKH receptors from *P. americana, A. pisum* and *P. humanus corporis* and the Diptera AKH receptors, except for *D. melanogaster* are most similar to the Hymenoptera AKH receptors. Of the none-insect receptors, only the receptors from *D. pulex* and from *C. gigas* have identities in the 30 and 40% range with the insect AKH receptors. All the invertebrate gonadotropine releasing hormone receptors (except the receptors form *D. pulex* and *C. gigas*) are only 20 to 30% identical to each other and all known AKH receptors.

### Phylogenetic Results

**Figure 4.**
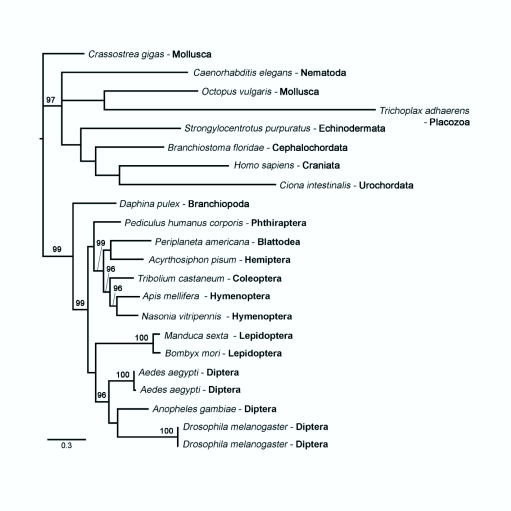
A 50% majority-rule consensus tree of 34,848 post burn-in trees from the Bayesian analysis, with Bayesian posterior probability percentage estimates above 90 are reported. The *scale bar* represents the estimated number of amino acid substitutions per sit). High quality figures are available online.

The Bayesian consensus tree (Figure 4) provides additional support that the cloned sequence is indeed the AHK receptor of *M. sexta.* The AKH receptor of *M. sexta* attaches to the tree with the only other known Lepidopteran sequence, *B. mori.* The tree topology also indicates that AKH receptor amino acid sequences are not evolving slowly enough to be phylogenetically informative for deep splits within the Insecta, however, they may be phylogenetically informative at the insect ordinal level. Sequences from different species within the same order form monophyletic groups.

### Expression of AKH receptor mRNA in fat body during development

**Figure 5.**
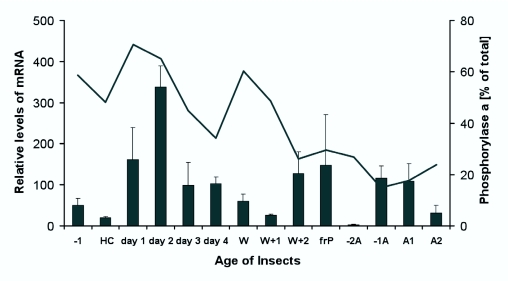
The relative level of the expression of mRNA for AKH receptor is shown as well as the maximal activation of GP by AKH (the curve of GP activity is redrawn from Ziegler 1984). The mRNA is presented as black bars, while the GP activity is expressed as a line. -1 is the last day of the 4^th^ instar, HC is the head capsule stage (molt), day I through day 4 of the last instar, is the feeding stage of the last instar, W is the wandering stage, frP is directly after pupation (cuticle still greenish) and A stands for adult, -2A stands for 2 days before adult emergence. High quality figures are available online.

Total RNA was isolated and cDNA was synthesized from fat body of larvae, pharate adults, and adults of different ages. qRT-PCR was conducted to measure the expression level of AKH receptor mRNA (Figure 5). This figure also includes data from Ziegler (1984), showing the level of GP activity following the injection of AKH (extract from one pair of CC from adults were used, which corresponds to about 20 pmol; this is much more than what is needed for a full effect of AKH (Ziegler 1990)). The maximal activation of GP corresponds quite well with the relative abundance of AKH receptor mRNA. The major difference we observed is at the wandering stage at the end of the fifth instar, when GP can be strongly activated while the mRNA is at a relatively low level.

Surprisingly, the mRNA level for the AKH receptor appears to be about four times higher in larvae of the second day of the fifth instar than in adults. In adult *M. sexta,* the maximal activation of GP by AKH is much less than in larvae, however, one has to keep in mind that in adults the main effect of AKH is not to induce the activation of GP, but to induce the mobilization of lipids (Ziegler et al. 1990).

### Expression of AKH receptor mRNA in larval fat body during starvation

**Figure 6.**
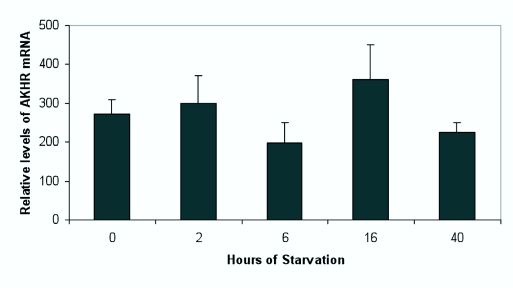
Relative level of the expression of mRNA for AKH receptor in larval fat body (late 2^nd^ day of the 5^th^ instar) after different times of starvation. There are some variations, however, there is no statistically significant difference between any two values. High quality figures are available online.

Starvation in larvae of *M. sexta* leads to the secretion of AKH and the activation of fat body GP. By 48 h of starvation, GP is inactivated again (Siegert 1987; Gies et al. 1988). We tested whether starvation has an effect on the expression of AKH receptor mRNA. Fat body of feeding larvae (late 2nd day of 5th instar) were compared with fat body from larvae of the same batch of larvae, which had been starved for two, six, sixteen or forty hours. No significant differences (Student's t-Test) were found between these starvation periods (Figure 6).

### Expression of AKH receptor mRNA in different tissues

**Figure 7.**
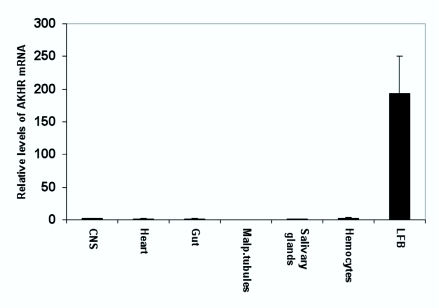
Relative level of the expression of mRNA for AKH receptor in different larval tissues. The larvae used were from late in the 2^nd^ day of the 5th instar. High quality figures are available online.

RNA was extracted from different tissues of larvae of the 2^nd^ day of the 5th instar and cDNA synthesized for qRT-PCR. The level of AKH receptor mRNA is very low in all tissues, except in fat body where significant levels of transcript were observed (Figure 7). In larvae, hemocytes had the second highest expression of AKH receptor, but still about 65 times lower than AKH receptor expression in the fat body. Other larval tissues have levels up to 475 times lower than the expression level in fat body.

**Figure 8.**
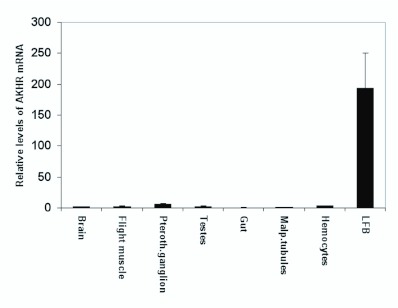
Relative level of the expression of mRNA for AKH receptor in different adult tissues (2^nd^ day of adult life) and for comparison in larval fat body. High quality figures are available online.

Tissues from two day-old adults showed a similar expression pattern, with significant levels of transcript present in fat body (Figure 8 and 5). In adults the highest level of AKH receptor mRNA, beside fat body, was found in the pterothoracic ganglion. However, the level was more than 30 times lower than in larval fat body. The next highest level was found in hemocytes, the level was about 55 times lower than in larval fat body.

## Discussion

Previously we characterized the AKH receptor of *M. sexta* with biochemical methods (Ziegler et al. 1995; Ziegler et al. 1998). After the recent identification of the AKH receptors from several insect species (Staubli et al. 2002; Park et al. 2002; Wicher et al. 2005; Kaufmann and Brown 2006, Belmont et al. 2006, Kaufmann et al. 2009), we designed degenerate primers based on evolutionary conserved amino acid residues of insect AKH receptors to isolate a full-length cDNA encoding a receptor with seven transmembrane segments from the fat body of *M. sexta,* which was considered to be an AKH receptor orthologue. It has strong sequence similarities with the AKH receptor of other insects, ranging from 84% identity with the receptor from *B. mori* to 39% identity with receptors from *Ae. aegypti* (Figure 3). Thus this is likely to be the AKH receptor of *M.* sexta.

With recent sequencing of insect genomes, several AKH receptors or AKH receptor-like proteins were identified. The identity with known AKH receptors was variable and it was not always clear whether a specific receptor was an AKH receptor. Some were therefore called AKH receptor-like (Kaufmann et al. 2009). A recent paper (Hansen et al. 2010) resolved the confusion by showing that there is a signaling system that up to now had not been recognized. Its function is not known. The ligands and the receptors are similar to AKH and corazonin, but distinct. So Hansen et al. called them AKH/corazonin-related peptides (ACP) and ACP receptors. The ACPs do not bind to AKH receptors or to corazonin receptors, nor do AKHs or corazonins bind to ACP receptors. In this discussion we will only consider the real AKH receptors. The phylogeny of the ACP and corazonin receptors was discussed before (Hansen et al.2010) and without knowing that some of these receptors are ACP receptors (Kaufmann et al. 2009).

The AKH receptors belong to the same protein family as the gonadotropin releasing hormone receptor. Staubli et al. (2002) pointed out that the AKH receptor is related to the human gonadotropin releasing hormone receptor. Gonadotropin releasing hormone and AKH receptors as well as the corazonin and ACP receptors all belong to a very large receptor family. They are found in vertebrates (reviewed by Tello and Sherwood 2009), insects, nematodes, crustaceans, mollusks, Echinodermata, primitive chordates, and possibly even the placozoan *T. adherens* (up to 23% identity with some insect AKH receptors). The *C.* *elegans* gonadotropin releasing hormone receptor-like protein has only a low identity (about 20%) with the insect AKH receptors or the human GnRH receptor. However, the ligand for the *C. elegans* receptor mobilizes in high concentration lipids in *Locusta americana* and carbohydrates in *P. americana* and the *D. melanogaster* AKH activates the *C. elegans* GnRH/AKH receptor (Lindemans et al. 2009).

The corresponding receptors of some invertebrates have up to 40% identity with the insect AKH receptors demonstrating the relatedness of these receptors. For example the Branchiopoda *Daphnia pulex* has a receptor that is 40% identical with the shorter splice variant of the AKH receptor of *Ae. aegypti.* All decapods have the same red pigment-concentrating hormone (RPCH) closely related to the AKHs of insects. *D. pulex* RPCH has a slightly different sequence and this peptide is surprisingly not able to induce the concentration of red pigment cells in a shrimp (Marco and Gäde 2010). In addition this RPCH from *D. pulex* mobilizes lipids in the stinkbug *Nezara viridula.* The function of this ligand in *D. pulex* is not known.

There is also a sequence for the tick, *Ixodes scapularis,* in GenBank (EEC14764.1), that has been called “arthropoda AKH receptor-like” (Kaufmann et al. 2009), however, the sequence is very short and contains only 4 transmembrane segments. This shows it is either an incomplete sequence or not a gonadotropin releasing hormone receptor-like sequence. Therefore it was not included in our survey.

The gonadotropin releasing hormone receptor of the Pacific oyster *Crassostrea gigas* is involved in the control of gametogenesis (Rodet et al. 2008), as gonadotropin releasing hormone receptors are in vertebrates. It has fairly strong identity with the insect AKH receptors up to 44% (Figure 3), while with the receptor from another mollusk, *Octopus vulgaris,* it is only 27% identical, and with all the other non-insects 34% or less.

Comparing these receptors shows that they are related, and have developed early in animal evolution. Some of them are distantly related, some of them closer, however, it is not possible to deduce the phylogenetic position of different groups of animals by comparing the relatedness of the gonadotropin releasing hormone receptors.

An old question in the AKH field is, is there one or more receptors. Many insects have more than one AKH, do they have also more than one AKH receptor? Based on biphasic response curves in bioassays there were speculations of more than one receptor (Gäde and Hayes 1995). In *M. sexta* we found that some inactive analogs increased the effect of low doses of AKH. Not knowing how to explain this we speculated that there might be a second receptor (Ziegler et al. 1998). We now think it much more likely that this is an allosteric effect. The genomes of *B. mori, A. mellifera, N. vitripennis, An. gambiae, A. pisum, P. humanus corporis,* and *T. castaneum* have been sequenced and only one receptor for each of these species was found. *D. melanogaster* and *Ae. aegypti* each have two splice variants of their AKH receptors, with extended C-terminals. It is not known whether these splice variants have different functions. In the catfish, *Clarias gariepinus,* deletion of 12 amino acids at the C-terminal reduced the internalization of the receptor (Blomenröhr et al. 1999). The insect receptors with different length of the C-terminal could have similar differences in internalization, but this should be experimentally tested. The present results, however, do not indicate that more than one AKH receptor is common. In summary it appears unlikely that many insects have more than one AKH receptor that is different from gonadotropin releasing hormone receptors in many other animals. Some insects have besides an AKH receptor, other proteins that are similar to gonadotropin releasing hormone receptors e.g., *T. castaneum* has two proteins like that, but they are not AKH receptors. One is an ACP receptor (EU138886), the other (XP_971565) does not appear to be an AKH receptor either, as it is a very large protein (974 amino acids) and has 10 transmembrane segments.

Previously, we demonstrated that AKH (also known as glycogen Phosphorylase activating hormone in *M. sexta*) mobilizes lipids from the fat body of adult *M. sexta* for flight (Ziegler and Schulz 1986) and in larvae it activates GP in fat body (Siegert and Ziegler 1983; Gies et al. 1988; Ziegler et al. 1990) during starvation and molting. During molting, insects starve (molting larvae of *M. sexta* are not able to feed 36 to 48 h) and this leads to a decrease in hemolymph glucose, which appears to trigger the secretion of AKH from the CC, keeping the trehalose levels high in the hemolymph by activating GP, which degrades glycogen (Gies et al. 1988). After 36 to 48 h of starvation GP is inactivated again. This could indicate that AKH is no longer secreted or that AKH receptor levels have strongly decreased. The expression of AKH receptor mRNA in fat body does not change during starvation. The AKH receptor appears to be continuously expressed, although after 40 h (when the larvae are on day 4 of the 5^th^ instar) expression might be decreasing (Figure 6), which would correspond to the decrease in the AKH receptor mRNA concentration with age (Figure 5). This is in agreement with results of Siegert (1988) who found that injected AKH activates GP, even after 48 h of starvation, although a bit less than during earlier times. These results together indicate that after about 48 hrs of starvation AKH is no longer secreted and so GP in fat body is inactivated.

In other insects AKH is also secreted into hemolymph during larval starvation, as was shown by Candy (2002) in *Schistocerca gregaria,* using antibodies to AKH. During starvation in *D. melanogaster* with AKH producing cells ablated, trehalose levels are much lower than in intact insects (Isabel et al. 2005), indicating that during starvation of intact animals AKH is secreted and mobilizes glycogen reserves to maintain high levels of hemolymph trehalose.

All the tissues we tested except fat body express AKH receptor mRNA at very low level, raising the question whether this gene might be continuously expressed at a low level or whether only a few cells in these tissues express AKH receptor (Figure 7 and 8). The highest level of expression found outside the fat body was in the pterothoracic ganglion of adults, 3.3% of the level in larval fat body. If AKH is injected into the pterothoracic ganglion close to nerve II N2a that innervates the third axillary muscle of the mesothorax (a muscle which is involved in flight steering), the muscle is strongly activated (Milde et al. 1995). Membrane preparations of the pterothoracic ganglion show some binding of AKH (Ziegler et al. 1995). These results together with the low expression of AKH receptor mRNA indicate that a pair of neurons or a few neurons in the pterothoracic ganglion have AKH receptors and respond to AKH.

Hemocytes of adults and larvae have AKH receptor mRNA expression 1.8 and 1.5% of larval fat body, respectively. That is lower than the expression level in the pterothoracic ganglion, but higher than in other tissues. If the AKH receptor is expressed in a minor subfraction of hemocytes, then AKH might have a role in these cells. In locusts it was shown that AKH enhances the immune response (Goldsworthy et al. 2003), so AKH could have a function in a subset of immune-responsive hemocytes.

Flight muscle, adult brain and larval central nervous system had 1.2, 1.1 and 1% of the amount of AKH receptor mRNA of larval fat body (one sample of brain RNA was slightly contaminated with DNA, so the amount of AKH receptor mRNA in brain is even lower than 1.1% of the amount in fat body). Flight muscle and adult brain was tested in a receptor binding assay (Ziegler et al. 1995) and no specific binding of AKH was found, so it is unlikely that AKH plays a role in these tissues. For the larval central nervous system it cannot be excluded that one or a few single cells respond to AKH, as was suggested for gustatory neurons that mediate sweet taste in *D. melanogaster* (Bharucha et al. 2008). Wicher et al. (2006) report AKH receptor mRNA in many tissues (salivary gland, ingluvies, Malpighian tubules, duodenum, ovaries, heart, flight muscle, brain, thoracic and abdominal ganglia, tracheae) of *P. americana.* As these are results obtained from RT-PCR and not from qRT-PCR it is not possible to directly compare them with our results. These authors have, however, used 40 cycles in their RT-PCR, so they have likely picked up very low concentrations of the receptor mRNA, possibly comparable to our results with tissues other than fat body. We do not think that AKH has a physiological function in these tissues.

When extracts of whole CC from adults were injected into larvae of different age of the last instar, all the experimental insects showed an increase in the activity of fat body GP (Ziegler 1984; Ziegler 1990). However, the magnitude of the response depended on the age of the larvae. The expression of mRNA for the AKH receptor changes similarly to the magnitude of GP activation. The main difference between the mRNA levels for the AKH receptor and the ability to activate GP was that the strong increase in the possibility of AKH to activate GP at the wandering stage showed no corresponding increase in mRNA. An exact correspondence of the AKH receptor mRNA level to the maximal activation of GP by AKH cannot be expected. We measured the activity of an enzyme (GP) that depends on signals from the receptor, not levels of the receptor protein. Therefore, other factors could also influence GP activity. In addition, mRNA may not be immediately translated into protein, and proteins can persist much longer than their corresponding mRNA. In the pharate adult (2A in Figure 5) the level of AKH receptor mRNA is very low. Correspondingly insects of this stage show only a very weak response to injected AKH (Ziegler 1984; Siegert 1996). This appears to be a time when AKH plays no role, or at the most a very small one.

Our results also demonstrate that AKH is important not only in adults, but in larvae as well, confirming our previous results (Siegert and Ziegler 1983; Ziegler et al. 1990). The levels of AKH receptor mRNA in larvae are clearly much higher than in adults, indicating the importance of AKH in larvae. Kaufmann and Brown (2006) and Groenke et al. (2007) also reported that expression of the AKH receptor is high in larval *An. gambiae* and *D. melanogaster* respectively.

The role of AKH to mobilize stored carbohydrate reserves in molt and starvation in larval stages, might actually have originated first in ancient insects, and the role of AKH in mobilizing energy stores for flight might have evolved only later after flight evolved. This hypothesis could be explored by characterizing AKH and AKH receptors in primitive, wingless insects.

## Abbreviations

**ACP**,: AKH/corazonin-related peptide
**AKH**,: adipokinetic hormone
**CC**,: corpora cardiaca
**GP**,: glycogen Phosphorylase
**qRT-PCR**,: Real Time PCR
**RPCH**,: red pigment concentrating hormone
